# Supplementary material for: Eight-year periodical outbreaks of the train millipede
Source: R Soc Open Sci. 2021 Jan 13;8(1):201399. doi: 10.1098/rsos.201399 (PMC7890500; doi:10.1098/rsos.201399)
Supplement: Supplementary Materials [file rsos201399supp1.pdf]

Supplementary Materials for:

Eight-year periodical outbreaks of the train millipede

Keiko Niijima, Momoka Nii, Jin Yoshimura

This PDF file includes the following:

1) Supplementary Text (Method Details)
2) Extended References (cited in Tables S1-S8)
3) Supplementary Tables S1-S8

Supplementary Text (Method Details)

Study points with reasons for selecting new points

The Mt. Yatsu site (figures 2*a*-*c*, tables S2, S6):

We had changed the main observation point from point m1 to point m2 in 1982, because numerous nymphs of the millipede in point m1 were dead in molting pouches in summer, 1980. Kokkaibashi (ko) was in an oak (*Quercus crispula*) forest, but the area was too small for a long-term survey. When there were no millipedes in the soil, new points were selected (m1 to te on Sep. 17-18, 1976 and m2 to sh on Oct. 17-18, 1983). Mt. Tennyo (te) was in a larch (*Larix caempferi*) plantation (planted in 1953) in Oizumi Village, Yamanashi Pref. and managed as the Prefectural Forest. Shishi-iwa (sh) was in shrubs, located ca. 500 m south of the point m2. Swarming and/or aggregating millipedes were observed at Yatsugatake Experiment Forest of Tsukuba University (y1, y2). Point y1 was in shrubs at an oak forest edge, next to a large drainpipe. Point y2 was in an oak forest with a path next to a public road. Moriyama (mo) was surveyed because many adult millipedes were observed in the fall of ~~Fall~~ 1983, when emergence of adult millipedes was one year earlier than the other sampling points in the Mt. Yatsu site.

The Yanagisawa site (figures 2*d*, 2*e*, tables S3, S7):

A beech forest (B) is on the northwest slope at an angle of ten degrees and 1,600 m a.s.l.; the forest consists primarily of 30- to 160 year-old beech (*Fagus crenata*) and 70- to 80-year-old oak. The vegetation of the oak forest (O) is similar to that of the beech forest, except there is no beech. A beech with fir (*Abies homelopis*) forest (F) is similar to the beech forest, aside from the addition of fir. Yokote Forest Road (Y), a section of approximately 300 m, was under construction; both side of the section are covered by deciduous broad-leaved forest. The invertebrates were surveyed at the point B in 1972-1987. In 1995-1998, the sampling point was changed to the oak forest (O) because the beech forest had been disturbed by repeated long-term surveys. These surveys were carried out by K. Niijima. The densities during the swarming period in the falls of 1980, 1988 and 1996 were

surveyed in the point F by 8, 36 and 10 people, respectively, instead of in the beech and oak forests in order to avoid disturbing the forests with too many people. The Yokote Forest Road (Y), especially a section under construction, was surveyed on October 20, 1980, because large-scale swarming of the millipede had been reported. The millipedes were also observed at the point Y on March 18, 1981, when the beech forest could not be accessed because of deep snow.

Extended References (cited in Tables S3, S5, S8)

37. Niijima K. 1974 The train millipede *Parafontaria laminata armigera* Verhoeff at Yanagisawa Pass. *Edaphologia* **9**, 17-21 (in Japanese).

38. Shinohara K, Terada M, Niijima K, Iwanami M, Yamamoto K, Ishii K, Takano M and Kuwabara Y. 1981 Report of observation of an outbreak of *Parafontaria laminata armigera* Verhoeff - at Daibosatsu and Yanagisawa Pass in September 1980 -. *Takakuwaia* **13**, 3-6 (in Japanese).

39. Iwanami M and Niijima K. 1981 The occurrence of *Japonaria laminata* (Attems)(Diplopoda) in the upper catchment area of Tama River. *Edaphologia* **24**, 39-45 (in Japanese).

40. Usuda K. 1989 Report of observation of an outbreak of the millipede. *The Journal of Biology Teacher's Association of Tokyo* **25**, 67-68 (in Japanese).

41. Niijima K and Shinohara K. 1997 Report on a meeting observing the train millipedes. *J. Biol. Teacher's Association of Tokyo* **33**, 41-42 (in Japanese).

42. Takakuwa Y. 1937 External Morphology of Train Millipede (*Fontaria armigera* Verhoeff). Journal of the Museological Society of Japan **35** (60), 211-216 (in Japanese).

43. Kiyosawa H. 1958 Myriapoda and Arachnida in Mt. Ontake and its surrounding areas. In Study on Mt. Ontake, Nature Part, **5** (Animals), 455-467 (in Japanese).

44. Hashimoto M, Kaneko N, Ito MT, Toyota A. 2004 Exploitation of litter and soil by the train millipede *Parafontaria laminata* (Diplopoda: Xystodesmidae) in larch plantation forests in Japan. *Pedobiologia* **48**, 71-81.

45. Kuwabara Y. 1993a Sequel to the swarming of the train millipede. *Takakuwaia* **25**, 22 (in Japanese).

46. Kuwabara Y. 1996b Myriapoda in Ogano town. In Nature of Ogano Town II. Fauna and flora in level ground and in mountainous district. pp. 165-177. Ogano Town, Saitama (in Japanese).

47. Nakamura O. 1988 Outbreak of millipedes. *Shizenshi Dayori* (= News from nature by Natural History Museum of Saitama) **8**, 8-9 (in Japanese).

48. Kuwabara Y. 1998 Records on myriapods collected in Ryogami and Otaki Villages. *Saitama Seibutsu* **38**, 32-33 (in Japanese).

49. Kuwabara Y. 1996a Records on the train millipede in Sotochichibu. *Saitama Seibutsu* **36**, 32 (in Japanese).

50. Kuwabara Y. 1992 Reports on the swarming of the train millipede. *Takakuwaia* **24**, 102 (in Japanese).
51. Kuwabara Y. 2001 Records on myriapods collected around Mt. Jomine Pass and Haccho Pass. *Saitama Seibutsu* (= Fauna and Flora of Saitama) **41**, 26-27 (in Japanese).
52. Kuwabara Y. 1985 Myriapod-fauna of Jumoji Pass and Mikuni Pass, Otaki Village, Chichibu County. *Saitama Seibutsu* **25**, 54-55 (in Japanese).
53. Kuwabara Y. 1993b Myriapod-fauna around Jumoji Pass, Okuchichibu. *Saitama Seibutsu* **33**, 43-44 (in Japanese).
54. Kuwabara Y. 2012 Records on the train millipede observed in 2011. *Saitama Dobutsu-ken Tsushin* (=Report of Saitama Animal Society) **71**, 13-16 (in Japanese).
55. Kuwabara Y. 2000 Myriapods of Ogawa Town. In Nature of Ogawa Town, Animal part (ed. Ogawa Town) pp. 321-328. Ogawa Town, Saitama (in Japanese).

Table S1. Study sites of *Parafontaria* species*.

	Study site (numbers from Figure S1)	Study period		Collected	Co-worker	Brood in
		Year	Date	species*		Table 3
**1**	Mt. Norikura and Mt. Ontake,	1985	2019/7/24	*P. l. a.*	K. Kobayashi	VII
	Gifu and Nagano Prefs.	1985	Oct. 7-10	*P. l. a.*	Y. Osumi; H. Matsumoto	
				P. l. a.		
				P. e.		
		1994	July 11-12	*P. e.*	Y. Saijo	
		2005	Oct. 21	*P. e.*	A. Kosaku	
		2009	Oct. 2-3	*P. l. a.*	M. Hasegawa	
				P. e.		
**2**	Terasawa Experiment Forest, Nagano Pref.	2005	Oct. 1-2	*P. l. a.*	T. Yoshida; T. Fujikawa	III
**3**	Mt.Nyugasa, Nagano Pref.	2016	Oct. 8-9	*P. l. a.*		VI, VII
**4**	**The Mt. Yatsu site:** Mt. Yatsugatake,	1974	May	*P. l. a.*	K. Ishii; M. Terada;	VI
	Nagano and Yamanashi Prefs.	-2016	Sep.		N. Kaneko; Y. Kuwabara;	Figs. 2, 7
					M. Iwanami; R. Uno;	
					M. Hashimoto; K. Fujita	
**5**	Kawakami Village, Nagano Pref. to	1982	Oct. 23	*P. l. a.*	M. Terada	V
	Ueno Village, Gunma Pref.	1983	Oct. 17-18	*P. l. a.*	Y. Kuroda; K. Ishii	
**6**	**The Yanagisawa site:** Yanagisawa Pass,	1972	May.	*P. l. a.*	K. Shinohara; K. Ishii;	II
	Yamanashi Pref, Saitama Pref. and	-2012	Sep.		Y. Kuwabara; M. Iwanami;	Fig. 3
	Tokyo Metropolis				K. Arimitsu; Y. Osumi	
**7**	Ogawa and Tokigawa towns, Saitama Pref.	2011	Oct. 29	*P. l. a.*	Y. Kuwabara	I
**8**	Hinoki Pass, Gujo City, Gifu Pref.	2006	Oct. 28	*P. e.*		
**9**	Mt. Nekodake, Ueda City, Nagano Pref.	1968	Aug. 2-3	*P. k.*	K. Shinohara	
		1984	Aug. 15		K. Shinohara	
**10**	Kayanotaira, Kijima Village, Nagano Pref.	2005	Sep. 12	*P. k.*	M. Kondo	
		2006	Sep. 10		M. Kondo	
		2008	Oct. 22		Y. Niijima	
**11**	Tokamachi City, Niigata Pref.	2006	Nov. 2-3	*P. k.*	T. Sawahata	
		2008	Sep. 10-11		M. Terada	
		2011	Oct. 26		S. Baba	
		2012	Sep. 18		J. Yoshimur	
**12**	Mt.Maruyama, Nikko City, Tochigi Pref.	1983	Oct. 21	*P. k.*	K. Ishii	
**13**	Mt. Fuji, Narusawa Village,	2011	Oct. 28	*P. l. l.*	Y. Hagiwara	
	Yamanashi Pref.	2016	June, 9		Y. Hagiwara	
**14**	Mt. Kiyosumi, Chiba Pref.	1974	2019/5/19	*P. l. l.*		
		1986	Mar. 14			

** P. e., P. echizenensis; P. k., P. kuhlgatzy; P. l. a., P. laminata armigera; P. l. l., P. laminata laminata.*

Table S2. Sampling procedure for collecting *P. laminata armigera* in the Mt. Yatsu site.

Hand sorting in forest soil									Nymphs extracted by the Tullgren apparatus.				
Sampling date			Life stage	Sampling procedure*			Point in Fig. 2b		Life stage	Sampling procedure			Extracted indivi-duals
Year	Month	Day		area in cm^2	depth in cm	repeti-tion				area in cm^2	depth in cm	repeti-tion	
1974	May	2-3	5th	30×20	35	1	ko						
	Aug.	5-6	5th	25×25	30	2	m1						
	Nov.	21	6th	25×25	25	2	m1						
1975	Nov,	22	7th	30×30	20	1	m1						
			7th	30×20	20	1	m1						
1976	July	6-7	7th	50×50	20	2	m1						
	Aug.	9-10	7th→A.	Qual.			m1						
			Adult	Qual.			m1						
	Sep.	17-18	Adult	50×50	15	5	te		2nd	100	0-4	1	0
	Nov.	8-9	Adult	Qual.			m1			100	4-8	1	4
1977	Sep.	27	1st	Qual.			m1			100	8-12	1	337
1978	Nov.	16-17	2nd	Tullgren			m1	→		100	12-16	1	285
1979	Aug.	19	3rd	Qual.			m1			100	16-20	1	126
1980	Aug.	30-31	(4th)	Qual.			m1			100	20-24	1	9
1982	Feb.	8-9	5th	10×10	20	2	m2			100	24-28	1	1
	Apr.	22-23	5th	Qual.			m2						
	Aug.	11	5th → 6th	Qual.			m2						
	Oct.	22-23	6th	25×25	30	1	m2						
1983	Aug.	5	6th→7th	25×25	0-5	4	m2						
			6th→7th	25×25	5-10	3	m2						
	Oct.	17-18	7th	25×25	20	2	sh						
	Dec.	8-9	7th	Qual.			m2						
1984	May	30	7th	25×25	20	5	m2						
	Aug.	1-2	7th				m2						
			7th→A.	25×25	20	5	m2						
	Aug.	17	Adult	Qual.			m2						
	Aug.	27-28	Adult	50×50	15	4	m2						
	Sep.	23	Adult	25×25	20	5	m2						
	Nov.	1-2	Adult	50×50	25	2	m2						
1985	May	7	Adult	25×25	15	5	m2						
	June	6	Adult	25×25	15	5	m2		1st	100	0-2	1	0
	July	11	Adult	25×25	15	3	m2				2-4		1
			Egg				m2			100	5-7	1	12
	Sep.	13	1st	Tullgren			m2	→			7-9		8
1994	Oct.	20-21	2nd	Tullgren			m2	→	2nd	25×25	0-5	2	0
2000	Apr.	24	7th	Qual.			te				5-10		6
	Sep.	28-29	Adult	Qual.			y2				10-15		1
	Oct.	27	Adult	Qual.			y2				15-20		1
	Nov.	30	Adult	50×50	35	1	y2						
2001	June	2	Adult	Qual.			y2						
2016	Sep.	17	Adult	Qual.			ka						

On road surface

Year	Month	Day	Life stage	area in cm^2	depth in cm	repeti-tion	Point in Fig. 2b
1984	Aug.	27	Adult	25×25		5	y1
	Sep.	24 21:00	Adult	50×50		30	m1
		25 8:00	Adult	50×50		50	m1

*The millipedes were collected by hand sorting except otherwise mentioned.
Qual.: Qualitative sampling.
Tullgren: The millipedes were extracted by using the Tullgren apparatus.

Table S3. Sampling procedure for collecting *P. laminata armigera* in the Yanagisawa site.

Hand sorting										Nymphs extracted by the Tullgren (Tul.) or Berlese (Ber.) apparatuses.					
Sampling date			Life stage	Sampling procedure*			Point in Fig. 3a	Reference**		Life stage	Sampling procedure				Extracted individuals
Year	Month	Day		area in cm^2	depth in cm	repetition					Apparatus	area in cm^2	Depth in cm	repetition	
1972	May	12	7th	50×50	40	1	B		→	7th	Tull.	20	0 - 5	13	0
	Aug.	3-4	7th→A.	50×50	45	4	B						5 - 10	9	0
	Nov.	7	Adult	Qual.			B						10 - 15	9	0
1973	Jan.	23	Adult	Qual.			B						15 - 20	4	0
	May	10	Adult	50×50	10	9	B	[37]	↘	7th→	Tull.	100	0 - 4	5	0
	July	1	Adult	50×50	10	4	B			Adult			5 - 9	2	0
			Egg										10 - 14	2	0
	Aug.	4	Adult	Qual.			B						15 - 19	1	0
			Egg										25 - 29	1	0
			1st	Tullgren			B						35 - 39	1	0
	Aug.	19	Adult	Qual.			B						40 - 44	1	0
			Egg						↘	1st	Tull.	100	Litter	3	0
	Sep.	16	1st	Tullgren			B						0 - 4	3	13
1974	June	2		Qual.			B		↘	1st	Tull.	100	0 - 4	5	0
1975	Nov.	23	3rd	Qual.			B						4 - 8	5	4
1977	May	17	4th	50×50	25	1	B								
	Oct.	8	5th	25×25	35	3	B								
1978	May	18	5th	Qual.			B								
1979	Dec.	13	7th	Qual.			B			7th	Ber.	25×25	0 - 5	3	0
1980	June	6	7th	25×25	30	2	B						5 - 20	1	0
	June	26-27	7th	25×25	40	3	B		→				20 - 25	1	0
	Aug.	25	Adult	25×25	25	6	B		→	Adult	Ber.	25×25	0 - 2	4	0
	Sep.	15	Adult	Qual.			B						2 - 4	4	0
	Sep.	30	Adult	25×25	10	12	F	[38]							
	Oct.	20	Adult	Qual.			Y	[39]							
	Dec.	9	Adult	25×25	45	4	B								
1981	Mar.	18	Adult	30×30	30	1	Y								
	June	8	Adult	25×25	35	5	B								
	Aug.	20	Adult	25×25	30	5	B								
			Egg	Qual.											
1982	June	18	1st	25×25	30	5	B								
	Aug.	18-19	2nd	25×25	30	5	B								
	Oct.	26-27	2nd	25×25	25	3	B								
1983	May	26	2nd	25×25	25	1	B								
	Aug.	11-12	2→3rd	Qual.			B								
	Sept.	21-22	3rd	Qual.			B								
1984	Oct.	12		25×25	10	5	B								
1985	July	12	4th	25×25	25	2	B								
	Aug.	23-24	4→5th	25×25	25	2	B								
			5th												
1987	Aug.	27	7th	25×25	15	3	B								
				50×50	15	2	B			6th	Ber.	25×25	Litter	6	0
1988	Oct.	9	Adult	25×25	10	42	F	[40]					0 - 5	5	0
1995	June	22-23	6th	25×25	20	6	O						5 - 10	5	0
	July	13-14	6th	25×25	30	6	O		→				10 - 15	5	0
1996	July	24	7th	25×25	15	6	O						15 - 20	2	0
	Oct.	6	Adult	25×25	10	16	F	[41]					20 - 25	1	0
1997	Aug.	20-21	Adult	50×50	10	3	O								
			Egg	Qual.					→	Egg	Ber.	qualitative survey			9
			1st	Berlese						1st					8
1998	Oct.	27, 30	2nd	Berlese			O		→	2nd	Ber.	25×25	0 - 5	5	0
2005	July	1	Adult	Qual.			F						5 - 20	5	67
2012	Sep.	23-24	Adult	Qual.									20 - 30	5	5

*Density is shown in Tables S6 (Mt. Yatsu site) and S7 (Yanagisawa site).

** Original sampling data of this paper, except for the cited referenes.

Table S4. Weather records (temperature and rainfall) near the study sites.

Point in Fig. S1 / Month	Mean temperature (°C)						Rainfall in mm				
	A	B		C	D	E	A	B	C	D	E
	Air	Air	—10cm	Air	Air	Air					
January	-3.2	-5.6	-0.7	-6.2	-3.0	1.3	103.4	38.0		51.7	30.3
February	-2.0	-4.7	-1.0	-6.6	-2.4	2.1	116.6	68.4		65.6	40.3
March	0.5	-2.1	0.2	-2.4	1.5	5.7	125.8	77.7		102.0	65.9
April	8.3	6.1	3.9	3.8	7.5	11.8	162.0	135.9		129.1	88.9
May	13.3	10.5	9.6	9.1	11.9	16.6	134.2	112.3		138.8	87.9
June	16.7	14.7	14.9	12.1	15.5	20.2	270.7	199.6		209.0	132.3
July	21.0	18.7	18.6	15.5	19.4	23.8	261.8	156.6		211.5	157.5
August	21.4	19.2	19.7	16.1	20.2	25.0	219.2	168.5		261.3	227.2
September	17.3	15.3	17.4	12.9	16.4	20.7	245.3	185.5		259.2	242.1
October	10.9	8.9	12.1	7.7	10.3	14.6	139.7	125.1		146.5	124.1
November	5.0	3.6	6.5	2.3	5.3	8.5	93.0	60.2		72.6	57.2
December	-0.2	-2.2	2.2	-3.2	0.0	3.4	113.7	43.0		44.3	19.0
Mean	9.1	6.9	8.6	5.1	8.6	12.8					
Total							1985.4	1370.8		1691.6	1262.3
Altitude in m	976	1350		1980	1120	232					
Survey years	(1968-1978)	(1970-1979)		(1997-2000)	(1957-1999)	(1971-2000)					
Latitude	36° 02'	35° 56'		35° 51'	35° 48'	35° 59'					
Longitude	137° 29'	138° 28'		138° 57'	138° 49'	139° 4'					

Weather data point

A: Kamigahora, Takane Town, Takayama City, Gifu Pref.

B: Nobeyama office of Tsukuba University, Minamimaki Village, Nagano Pref.

C: Kumotori Mountain Hut, Otaki Village, Saitama Pref. (Upper limit of the train millipede).

D: Ochiai Branch, Bureau of Waterworks, Tokyo Metropolitan Government, Koshu City, Yamanashi Pref.

E: Chichibu City, Saitama Pref. (Low elevation; no train millipede).

Note.: Weather data for A, C and E are data source from Japan Meteorological Agency.

Those for B and D are described in the study site.

Table S5. Records of train obstructions by the millipede.

Year	Month	Day	Obstructed section (stations# between)	Species identification*1	References, JR documents*2, Newspapers*3
**The Chuo Line, Shiojiri City (S) and Kamiina-gun (K), Nagano Pref.**					
1920	Fall		Niekawa-Narai	OK	[6]
1928	Fall		Ono-Shiojiri	OK	[6]
1936	Sep.	5-21	Niekawa-Kisohirasawa	OK	JR1, JR2;
	Oct.	7-12	Hideshio-Niekawa		[42]
1976	Oct.	14	Ono-Shiojiri	No spec.	NP1
**The Koumi Line, Hokuto City, Yamanashi Pref. (Y) and Minamimaki-gun, Nagano Pref. (N)**					
1936	Sep.	10-15	Kaioizumi-Nobeyama	OK	
		16	Kaioizumi-Kiyosato		JR1, JR2;
		22-27	Kiyosato-Nobeyama		[42]
	Oct.	7-12	Kaioizumi-Kiyosato		
			Kaioizumi-Nobeyama		
1943	Sep.-Oct.		Shinanokawakami-Sakuuminokuchi	No spec.	JR3
1952	Oct.	10	Kaioizumi-Nobeyama	OK	NP2. [7]
1960	Oct.	6	Kiyosato-Nobeyama	No spec.	NP3. JR3, [7]
1968	Sep.-Oct.		Kaikoizumi-Nobeyama	No spec.	JR3
1976	Sep.-Oct.		Kaikoizumi-Nobeyama	OK	JR3
1984	Sep. -Oct,		Kaikoizumi-Nobeyama	OK	JR4

#: Location of the stations

Station	Altitude (m)	Latitude	Longitude
Ono (K)	813	36º03′	137º58′
Shiojiri (S)	716	36º03′	137º57′
Hideshio (S)	813	36º03′	137º54′
Niekawa (S)	872	36º01′	137º52′
Kisohirasawa (S)	914	35º59′	137º50′
Narai (S)	934	35º58′	137º49′
Kaikoizumi (Y)	1,044	35º52′	138º22′
Kaioizumi (Y)	1,159	35º54′	138º25′
Kiyosato (Y)	1,275	35º55′	138º26′
Nobeyama (N)	1,346	35º57′	138º28′
Shinano-kawakami (N)	1,136	35º59′	138º32′
Saku-uminokuchi (N)	1,040	36º01′	138º29′

*1: Species identifications.
OK: Samples were identified by the specialsts of millipedes.
No spec.: Records without specimens.

*2: Internal documents of the Japan National Railway.
JR1: M. Kasai M. 1936. Millipedes causing the slipping of wheels of rolling stock. *Research Records on Operations of Institute of the Minister's Secretariat of Railways* (by Research Office, Japanese Government, Tokyo). **24** (31), 7 pages (in Japanese with English summary).
JR2: Kaze K. 1938. On the millipedes threatening the Koumi Line operations. *Meeting Record of 25th Lecture of Railroad Maintenance* (in Japanese).
JR3: Nagano Railroad Administration. 1976. The train millipede on the Koumi Line. *Nagano Railroad Administration*. 30pp (in Japanese).
JR4: Ikeda T. 1984. Train damage situations accompanyed with the emergence of train millipedes on Koumi Line. 12pp (in Japanese).

*3: Japanese Newspaper.
NP1: The Asahi Shinbun. Millipedes attacked Chuo Line, too: between Higashi-shiojiri and Shiojiri. (October 15 morining edition, 1976).
NP2: Mainichi Shinbun. Millipedes stopped trains, but the cause of mass attack yet unknown (written by Y. Takakuwa.). (October 14, 1952).
NP3: The Asahi Shinbun. A horde of millipedes stopped trains in Koumi Line. (October 7 morning edition, 1960).

Table S6. Life stage, activities, distribution in soil and density for *P. laminata armigera* in the Mt. Yatsu site.

In forest soil

Sampling date			Life stage	Activity		Collected individual	Distribution in soil (%) (depth in cm)				Density (individual/m^2)
Year	Month	Day					0-5	5-10	10-15	15-20	x ± S.D.
1974	May	2-3	5th	hibernation	↑	28	0	3.6	39.3	57.2	467
	Aug.	5-6	5th	active		488	6.8	41.2	26.8	25.2	3904
	Nov.	21	6th	hibernation		117	0	45.3	44.4	10.3	936
1975	Nov,	22	7th	hibernation		34					378
			7th	hibernation	1976 generation	17					283
1976	July	6-7	7th	feeding		123					246
	Aug.	9-10	7th→A.	molt		6					
			Adult	molted		7					
	Sep.	17-18	Adult	outbreak		85					68 ± 104
	Nov.	8-9	Adult	before hibernation	↓	20					
1977	Sep.	27	1st	active	↑	19					
1978	Nov.	16-17	2nd	hibernation		762	0	44.7	37.4	17.9	
1979	Aug.	19	3rd	molted		54					
1980	Aug.	30-31	(4th)	(active)		0					
1982	Feb.	8-9	5th	hibernation		35	0	11.4	68.6	20.0	1,750
	Apr.	22-23	5th	hibernation		60					
	Aug.	11	5th → 6th	molt		113					
	Oct.	22-23	6th	active		95	0	32.6	61.1	6.3	1520
1983	Aug.	5	6th→7th	molt		5					20
			6th→7th	molt	1984 generation	14					75
	Oct.	17-18	7th	active		53	0	83.0	17.0	0	424
	Dec.	8-9	7th	hibernation		90					
1984	May	30	7th	active		147	3.1	52.7	41.9	2.3	470 ± 596
	Aug.	1-2	7th	before molt		1					
			7th→A.	molt		74	0	72.0	26.7	1.3	240 ± 303
	Aug.	17	Adult	molted		8					
	Aug.	27-28	Adult	swarm		361					361 ± 599
	Sep.	23	Adult	outbreak		78	47.5	51.3	0	1.3	250 ± 206
	Nov.	1-2	Adult	hibernation		84	2.4	40.5	48.8	8.3	168
1985	May	7	Adult	after hibernation		142	55.6	44.4	0		454 ± 58
	June	6	Adult	copulate, feeding		128	26.8	72.4	0.8		410 ± 55
	July	11	Adult	oviposit, copulate	↓	49	46.9	36.7	12.2	4.1	261 ± 65
			Egg		1992 generation	9643	27.2	71.1	1.7	0	51429 ± 1704
	Sep.	13	1st	active		21	4.8	95.2			
1994	Oct.	20-21	2nd	active		8	0	75.0	12.5	12.5	
2000	Apr.	24	7th	hibernation		2					
	Sep.	28-29	Adult	active	2000 generation	>85					
	Oct.	27	Adult	active		>59					
	Nov.	30	Adult	hibernation		40	0	5.0	20.0	75.0	160
2001	June	2	Adult	copulate, feeding		>300					
2016	Sep.	17	Adult	swarm	2016 generation	12					

On road surface

Year	Month	Day	Life stage	Activity		Collected individual	0-5	5-10	10-15	15-20	Density
1984	Aug.	27	Adult	swarm		240					768 ±320
	Sep.	24 21:00	Adult	swarm	1984 generation	118*					16 ±7
		25 8:00	Adult	swarm		1435*					115 ±50

* Observed only (not collected).

Table S7. Life stage, activity, distribution in soil and density for *P. laminata armigera* in the Yanagisawa site.

Sampling date			Life stage	Activity	Generation	Collected individual or batch*	Distribution in soil (%) (soil depth in cm)				Density (individuals/m^2)
Year	Month	Day					0-5	5-10	10-15	15-25	x ± S.D.
1972	May	12	7th	active	↑	23					92
	Aug.	3-4	7th→A.	molt		9					9 ± 10
	Nov.	7	Adult	prepare for hibernation		24					
1973	Jan.	23	Adult	hibernation	1972 generation	63					
	May	10	Adult	swarm, copulate		87					39 ± 39
	July	1	Adult	lay eggs and dying		27					28 ± 14
			Egg	300-500 eggs in a mass		12*					12* ± 11
	Aug.	4	Adult	majority dead	↓	2					
			Egg	just before hatch	↑	ca. 500					
			1st	just hatched		13					
	Aug.	19	Adult	no adults		0					
			Egg	just before hatch		ca. 750					
	Sep.	16	1st	active		4					80 ± 180
1974	June	2		not found							
1975	Nov.	23	3rd	hibernation		29					
1977	May	17	4th	inactive		108					432
	Oct.	8	5th	active	1980 generation	117	4	28	37	31	624 ± 403
1978	May	18	5th	inactive		25					
1979	Dec.	13	7th	hibernation		52					
1980	June	6	7th	active		58					547 ± 421
	June	26-27	7th	active		114	0	←	100	→	
	Aug.	25	Adult	active		274	4	←	96	→	267 ± 229
	Sep.	15	Adult	creep on soil surface		14					
	Sep.	30	Adult	swarm, rest in litter		175					235 ± 547
	Oct.	20	Adult	outbreak		38					
	Dec.	9	Adult	hibernation		31	3	←	97	→	124 ± 80
1981	Mar.	18	Adult	hibernation		27					300
	June	8	Adult	copulate		121	78	←	22	→	102 ± 40
	Aug.	20	Adult	no adults	↓	0					0
			Egg	just before hatch	↑	2129	100	0	0	0	
1982	June	18	1st	active		69	0	25	← 75	→	221 ± 2450
	Aug.	18-19	2nd	just after molt		71	0	7	← 93	→	266 ± 78
	Oct.	26-27	2nd	active		134	0	6	68	26	715 ± 1050
1983	May	26	2nd	active		39	0	0	51	49	624
	Aug.	11-12	2→3rd	molt		30					
	Sept.	21-22	3rd	active	1988 generation	5					
1984	Oct.	12		not found		0					
1985	July	12	4th	active		24	0	0	54	46	192
	Aug.	23-24	4→5th	molt		2					16
			5th	just after molt		138	0	1	62	37	1104
1987	Aug.	27	7th	active		5					30 ± 17
						17					
1988	Oct.	9	Adult	creep on soil surface	↓	>80					103
1995	June	22-23	6th	active		102	55	43	2	0	272 ± 448
	July	13-14	6th	active	1996 generation	211	4	82	11	3	563 ± 562
1996	July	24	7th	active		38	0	58	42		101 ± 179
	Oct.	6	Adult	creep on soil surface		86					86 ± 52
1997	Aug.	20-21	Adult	no adults		0					0
			1st	just after hatch	2004 generation	8					
1998	Oct.	27, 30	2nd	preparefor hibernation		72	0	33	58	9	230 ± 176
2005	July	1	Adult	active		41					
2012	Sep.	23-24	Adult	active	2012 generation	32					

Table S8. Distribution records of all the confirmed broods (Broods I-VII, no confirmed record for VIII) of *P. laminata armigera* .

S8.1. Brood VI (1976 ± 8n Generation: 1920, 1928, 1936, 1952, 1960, 1968, 1976, 1984, 1992, 2000, 2008, 2016)**

Prefcture / County (-gun) or City (C.) / Village (V.) or Town (T.)	Sampling date Year	Month	Day	Collected individual	Vegetation*	Altitude (m)	Latitude (° ')	Longitude	Literature or collector*
Nagano Pref.									
Shiojiri C.									
Takabochi	1976								[9]
Shiojiri Pass	1976	Sep.-Oct.		24		1,000	36.05	138.02	[9]
Mt. Higashiyama	1984	Sep.-Oct.				1,400	36.07	138.02	M. K.
Kataoka	1985	May	7	2		1,000	36.09	138.00	[9]
Kakenoyu	1976	Oct.							
Kiso-gun									
Nezamenotoko, Agematsu T.	1952	Oct.	9-10			720	35.46	137.42	[43]
	1976	Oct.							[11]
Kamiina-gun									
Tatsuno T.	1984	Sep.-Oct.				1,000	36.01	137.56	M. K.
Minowa T.	1976	Oct.				900	35.55	137.56	[11]
	1984	Sep.-Oct.							M. K.
	1992	Nov.	13	16					M. O.
Kayano highland, Minowa T.	2005	Oct.	2	6	Db. F.	1,000-1,200	35.54	138.02	K. N.
Gonbe Pass, Minamiminowa T.	1984	Sep.-Oct.				1,300	35.52	137.52	M. K.
Matsumoto City									
Utsukushigahara, Sanjiro Farm and	1976	Oct.	4	11	*L. k* . P.	1,350-1,700	36.11-13	138.07-08	[15]
Tobira Pass	1984	Fall							
Mt. Hachibuse	1976	Oct.	5		Grass.	1,928	36.10	138.04	[9]
Suwa City									
Kirigamine Highland	1976	Oct.	1			1,200-1,700	36.05	138.09	[15]
	1984	Sep.	18	6	*L. k.* P.				Oth.
Ikenokurumi	1984	Sep.	18			1,558	36.05	138.10	Oth.
Kirigamine Farm	1984	Sep.	18			1,318	36.04	138.10	Oth.
Tatenoumi	1984	Sep.-Oct.				1,100-1,600	36.05	138.08	M. K. *et al* .
Suwa-gun									
Sanrigahara, Fujimi T.	1984	Sep.-Oct.				1,200	35.55	138.18	M. K.
Chino C.									
Fujimidai-Kurumayama	2000	Sep.	15	12	*L. k.* P.	1,600-1,700	36.06	138.12	M. O.
Near Daimon Pass	1984	Sep.-Oct.				1,350-1,810	36.07	138.14	M. K. *et al* .
Shirakaba Lake	1953	July	26	1		1,500	36.06	138.14	Oth.
	1984	Sep.	1						[9]
Kitasaku-gun									
Mt. Tateshina, Vinus Line,	1976	Fall			*L. k.* P.	1,600-1,700	36.06	138.16	[15]
Tateshina T.	1984	Aug.	7						[15]
Sanrigahara, Fujimi T.	1984	Sep.-Oct.							
Saku C.						1,200	35.56	138.17	M. K.
Kyowa farm	1984	Sep.-Oct.			*L. k.* P.	1,300	36.10	138.18	M. K.
	2000	Oct.	27	28		1,400			Oth.
Minamisaku-gun									
Yachiho Highland, Sakuho T.	1976	Fall				1,200	36.07	138.25	[15]
	1984	Fall			*L. k.* P.	1,100-1,800			[15]
Sakuho T.	1985	May	14	21	*L. k.* P.	1,500	36.07	138.26	Oth.
Uminokuchi, Minamimaki V.	2000	Fall		9	*L. k.* P.	1,300	36.01	138.28	M. K. *et al* .
Kawakami Exper. Forest Tsukuba Univ.	2000	Sep.	27	12	*L. k* . P.	1,600	35.55	138.30	K. N.
Nobeyama	1992	Fall							[23]
	2000	Oct.	8	20	*Q. c.* F.	1,345	35.57	138.28	Oth.
	2008	Fall							[23]
Yamanashi Pref. (Oizumi T., Hokuto C.)									
Kannondai	2000	Oct.			*L. k.* P.	1,360	35.55	135.20	[44]
	2016	Sep.	17	12					M. H., K. N., J. Y.
Mt. Tennyo	1976	Sep.	17	85		1,500	35.55	138.24	K.N.
	2000	Fall							[44]
Kiyosato Highland	1977	July	22	8		1,400-1,500	35.56	138.25	[9]
Mt. Utsukusinomori	1992	Aug.	27-28			1,500	35.56	138.25	[45]
	2000	Sep.-Oct.		401					Oth.

*Abbreviations are listed in the last table.

** The records in the Mt. Yatsu site and the train obstruction records by the millipede along the Chuo and Koumi Lines are shown in figure 4 and tables S5-S6.

S8.2. Brood II (1972 ± 8n Generation: 1956, 1972, 1980, 1988, 1996, 2004, 2012)**

Prefecture	County (-gun) or City (C.)	Village (V.) or Town (T.)	Sampling date Year	Month	Day	Collected individual	Vegetation*	Altitude (m)	Latitude (° ')	Longitude	Literature or collector*
Yamanashi Pref.											
	Hokuto City										
		Ogawayama Forest Road	1980	Fall			*L. k.* P.	1,450	35.54	138.34	K. Y.
			1988	Oct.	19						K. Y.
			1996	Sep.	26						K. Y.
		Fujimidaira, Mizukaki Lodge	1980	Fall			*B.* spp. F.	1,530-1680	35.53	138.35	K. Y.
			1988	Oct.	19						K. Y.
			1996	Sep.	26						K. Y.
		Tokusa Pass	1988	Oct.	10		*L. k.* P.	1,450	35.50	138.34	K. Y.
			1996	Sep.	26						K. Y.
	Yamanashi City (Mitomi)										
		Nishizawa Valley	1980	Fall			*B.* spp. F.	1,750	35.52	138.43	K. Y.
			1988	Oct.	2						K. Y.
			1996	Sep.	11						K. Y.
		Kari pass	1980	Oct.	25-26	12		1,780	35.52	138.49	[38]
		Hirose	1980	Fall			*B.* spp. F.	1,100	35.51	138.46	K. Y.
			1988	Oct.	2						K. Y.
		Mt. Kentoku	1972	Oct.	1	2		1,800	35.49	138.43	[11]
		Nakatsu Shrine	1980	Fall			*Cr. j.* P.	1,000	35.49	138.45	K. Y.
			1988	Oct.	2						K. Y.
	Koshu City										
		Mt. Ishiyasudo	1980	Oct.	25-26	35		1,673	35.50	138.48	[38]
		Inukiri Pass	1980	Oct.	25-26	24	*L. k* . P.	1,200	35.49	138.50	[38]
		Ichinose-takahashi	1980	Oct.	25-26	11	*B.* spp. F.	1,450	35.49	138.49	K. Y.
			1988	Oct.	2						K. Y.
			1996	Sep.	11						K. Y.
		Mikubo Highland	1980	Fall			*B.* spp. F.	1,500	35.47	138.47	K. Y.
			1988	Oct.	2						K. Y.
			1996	Sep.	11						K. Y.
		Saiki Forest Road	1980	Oct.	21		*Q. c* F.	1,300-1,400	35.47	138.48	[39]
		Gorota	1980	Sep.	30	178	*L. k* . P.	1,150	35.45	138.49	[38]
		Sensuidani	1980	Fall			*Q. c* . F.	1,100-1,200	35.47	138.52	[39]
		Marukawa Pass-Sakeishi	1980	Sep.	29	72	Db. F.	1,400-1,650	35.45	138.49	[38]
		Sakeishi, Enzan	1980	Sep.	29	231	*L . k.* P.	950-1,000	35.44	138.48	[38]
		Mt. Daibosatsu	1980	Nov.	6	36	Conif. F.	1,600-1,700	35.46	138.51	[38]
			1988	Oct.	2						K. Y.
			1996	Oct.	10			1,856			K. Y.
		Toyama	1988	Oct.	2			1,605	35.44	138.50	K. Y.
			1996	Sep.	11						K. Y.
	Kitatsuru-gun (TabayamaV.)										
		Aoiwa Cave and	1956	Oct.	2-3		*Q. c.* F.	1,200	35.50	138.55-56	H. T.
		Sanjonoyu Spa	1980	Fall							K. Y.
			1988	Oct.	16						K. Y.
			1996	Sep.	11						K. Y.
		Nanatsuishi Hut	1980	Fall			*Q. c.* F.	1,600	35.50	138.58	K. Y.
			1988	Oct.	16						K. Y.
			1996	Sep.	11						K. Y.
Saitama Pref.											
	Chichibu-gun (Ogano T.)										
		Mt. Futago	1989	Aug.	2	7	*Cr. j* . P.	700-950	36.04	138.52	[46]
	Chichibu City										
		Experiment forest of Tokyo	1988	Oct.	26	36	*Cr. j., L. k.* P.	700	35.56	138.50	[47]
		University, Irikawa, Otaki	1997	Aug.	2-3	7		800	35.57	138.51	[48]
		Terai	1996	Oct.	31	4		700	35.57	138.54	Oth.
		Mitsumine Shrine	1980	Fall				1,100	35.56	138.56	38]
			1981	May	10	50					[9]
		Ochi River	1988	Fall				700	35.55	138.58	[47]
		Mt. Kirimogamine	1972	Oct.	30	92	Db. F.	1,523	35.54	138.57	[9]
Tokyo Metropolis											
	Nishitama-gun (Okutama T.)										
		Mt. Takanosu	1972	Sep.	23-24			1,560-1,737	35.50	139.01	[39]
			1981	July	22	2					[9]
		Mt. Mutsuishi	1981	May	10	11		1,479	35.49	139.03	[9]
			1997	June	22	18	*Q. c* . F.	1,350			N. M.
		Mt. Kumotori	1956	Oct.	2-3			2,000	35.51	138.57	H. T.
			1980	Fall			Db. F.	1,900			[39]

*Abbreviations are listed in the last table.

**The records in the Yanagisawa site are shown in table S7.

S8.3. Brood V (1975 ± 8n Generation: 1935, 1943, 1975, 1983, 1991, 1999, 2007)									
Prefecture	Sampling date			Collected	Vegetation*	Altitude	Latitude	Longitude	Literature
County (-gun) or City (C.)				individual					or
Village (V.) or Town (T.)	Year	Month	Day			(m)	(º	')	collector*
Nagano Pref.									
Matsumoto C.									
Shimizu highland	1991	Sep.	20	16	*L. k* . P.	1,200-1,300	36.09	137.51	M. K., M. O.
Tobira Pass	1975	Oct.			Db. F.	1,600	36.11	138.08	[15]
Sanjiro Farm, Iriyamabe	1983	Sep.	15		*L. k.* P.	1,350	36.12	138.06	[15]
Funagasawa, Iriyamabe	1991	Oct.	3	7	*L. k.* P.	1,400	36.14	138.05	M. O.
Border of Okaya C.	1991	Sep.	20	2	*L. k.* P.	1,850	36.10	138.04	M. K., M. O.
Shiojiri C.									
Kosobu	1983	Sep.	9	24	Conif. F.	1,100	36.05	137.53	[9]
Mt. Takabocchi, Kataoka	1975	Fall			*L. k* . P.	1,500-1,600	36.08	138.02	[11]
	1983	Fall							[15]
	1991	Sep.	20						M. K., M. O.
Gakenoyu Spa, Kataoka	1975	Fall				1,000	36.09	138.01	M. K., M. O.
Suwa-gun									
Wada Pass and Nanashima-	1975	Fall			*L. k* . P.	1,080-1,660	36.07-09	138.09-10	[14]
yashima Swamp Simosuwa T.	1983	Oct.							[14]
	1991	Fall							[23]
	1999	Fall							[23]
	2007	Fall							[23]
Suwa**	1935	Fall							[21]
Minamisaku-gun									
Kaize, Sakuho T.	1991	Oct.	16	3	*L. k* . P.	800	36.09	138.30	M. O.
Furuya Dam, Sakuho T.	1991	Nov.	2	18		1,000-1,100	36.08	138.36	[49]
Jukkoku pass, Sakuho T.	1991	Nov.	2	110	*L. k* . P.	1,340-1,360	36.06	138.39	[50]
	2000	Aug.	2	16	*Q. c* . F.				[51]
Koumi-Kogen Golf, Koumi T.	1983	Oct.	17	4	*L. k* . P.	1,100	36.03	138.29	K. N., K. I.
Oyasawa, Koumi T.	1983	Oct.	17	87	Db. F.	900	36.05	138.32	K. N., K. I.
Kawadaira, Minamimaki V.									
Shiroiwa, Kitaaiki V.	1983	Nov.	2			1,100-1,300	36.04	138.36	[50]
Moriyama, Kawakami V.	1983	Oct.	17	169	Db. F.	1,000-1,300	35.60	138.31	K. N., K. I.
Gunma Pref. (Ueno V., Tano-gun)									
Mt. Tenbo	2000	Aug.	3		*L. k* . P.	1,260	36.06	138.39	[51]
Budo Pass	1983	Oct.	18	8	Db. F.	1,400-1,540	36.04	138.39	K. N., K. I.
	1991	Oct.	2-4		*L. k.* P.				Oth.
	2000	Aug.	2	10	*L. k* . P.				[51]
Mt. Suwa	1992	Aug.	15			1,000	36.03	138.44	Oth.
Kurumidaira	1983	Oct.	18			700	36.03	138.48	H. T.
Saitama Pref. (Chichibu C.)									
Hacho Tunnel	1991	Oct.	9		Db. F.	1,250	36.02	138.50	[50]
Hacho Pass, Nakatsugawa	2000	Aug.	3	8		1,490	36.02	138.50	[51]
Akuishi, Mikuni Ridge	1984	Aug.	3	1		1,840	35.59	138.43	[52]
Benkeiiwa, Jumoji Pass	1992	Aug.	4	1		1,850	35.57	138.44	[53]
Tokyo Metropolis (Okutama T., Nishitama-gun)									
Osawairi, Kotozura	1983	Sep.	23	25	Db. F.	700	35.48	139.05	[9]
Mt. Mitake-Mt. Otake	1967	Sep.	15	1	Db. F.	900-1,200	35.46-47	139.08-09	[9]

*Abbreviations are listed in the last table.

**Type locality.

S8.4. Brood VII (1977 ± 8n Generation: 1977, 1985, 2001, 2009)

Prefecture / County (-gun) or City (C.) / Village (V.) or Town (T.)	Sampling date: Year	Month	Day	Collected individual	Vegetation*	Altitude (m)	Latitude (º ')	Longitude (º ')	Literature or collector*
Gifu Pref.									
Gero C. (Osaka T.)									
Suzuran Pass (37ha)	2010	July, Aug.	2, 6	12	*C. j.* P.	1,200	35.58	137.21	H. M., Y. M.
Mt. Ohira (36yo, 43nu)	2010	July, Aug.	2, 6	11	*C. j.* P.	1300-1400	35.58	137.21	H. M., Y. M.
Osaka T. (46u)	2010	July	2		*C. j.* P.	1,300	35.56	137.21	H. M., Y. M.
Osaka T (56ri)	2010	July, Aug.	5, 9	34	*C. j.* P.	1,200	35.57	137.24	H. M., Y. M.
Takayama C. (Takane)									
Toriya Pass	2009	Oct.	3	9	Db. F.	1,350	36.01	137.25	M. H., K. N.
Konohara Highland	2009	Oct.	3	3	Conif. F	1,600	36.05	137.29	M. H., K. N.
Takane Dam	1977	Sep.-Oct.							[22]
	1985	Oct.	9	50	*Q. c.* F.	1,100	36.02	137.29	K. N., Y. O.
Nomugi Pass	1985	Oct.	9	28	*B.* spp. F.	1,460	36.03	137.36	K. N., Y. O.
Tomenohara	1985	Oct.	10	47	*Q. c.* F.	1,210	35.59	137.32	K. N., Y. O.
Nagano Pref.									
Matsumoto C.									
Shirahone Spa, Azumi	2001	Oct.	6	3		1,600	36.09	137.37	T. F.
Kanayama, Azuni	1985	Oct.	7-8	40	*Q. c.* F.	1,450	36.08	137.37	K. N., Y. O.
Ushidome Pond, Azumi	2001	Sep.	25	6		1,560	36.07	137.37	T. Y.
Suzuran, Mt. Norikura, Azumi	1977	Oct.		8	*L. k.* P.	1,200	36.07	137.37	[15]
	1985	Sep.	28	6	*L. k.* P.	1750-1550			Oth.
Inakoki, Azumi	1977	Sep.-Oct.				900	36.09	137.46	[15]
	1985	Oct.	7-8	25	*L. k.* P.	1,100			K. N., Y. O.
Shirahone	2001	Sep.	22	3		1,600	36.09	137.38	T. F.
Yasudaira, Nakawa	1985	Oct.	9	13	*L. k.* P.	1,100	36.03	137.40	K. N., Y. O.
Kiso-gun.									
Nagamine Pass, Kiso T.	1985	Oct.	9			1,340	35.58	137.33	K. N., Y. O.
Nishino, Kiso T.	2009	Oct.	3	3	*L. k.* P.	1,200	35.58	137.35	K. I.
Kuzo Pass, Tabanosawa, Kiso T.	1985	Oct.	9	15	*L. k.* P.	1,250	35.56	137.35	K. N., Y. O.
Mt. Ontake, Otaki V.	1985	Sep.	17	10	*L. k.* P.	1,000-1,600	35.52-53	137.29-34	Oth.
Ontake grabe site, Kiso T.	2001	Oct.	20	2					T. F.
Ontake Hakkaisan Shrine, Kiso T.	2009	Oct.	2	5	*L. k.* P.				M. H., K. N
Misogawa Dam, KisoT.	2009	Oct.	30	5		1,150	36.00	137.46	Oth.
Upper reaches of Denjo River, Otaki V.	1986	July	24	6	Conif. F.	1,800	35.52	137.29	Oth.
Suzugasawa, Kiso T.	1985	Oct.	9	10	*Q. c.* F.	1,160	35.49	137.31	K. N., Y. O.
Kamiina-gun (Minamiminowa V.)	2001	Oct.	11	4		1,500	35.52	137.51	T. F., T. Y.
Shizuoka Pref. (Aoi-ku, Shizuoka C.)									
Fujimi Pass	2001	Oct.	27	10		1,180	35.11	138.15	T. F.
Ikawa Dam	2001	Oct.	27			800	35.12	138.13	

*Abbreviations are listed in the last table.

S8.5. Brood I, III, IV

S8.5.1. Brood I (1971±8n Generation: 1979, 1995, 2011)

Prefecture / County (-gun) or City (C.) / Village (V.) or Town (T.)	Sampling date: Year	Month	Day	Collected individual	Vegetation*	Altitude (m)	Latitude (° ')	Longitude	Literatures or collectors*
Saitama Pref.									
Chichibu City									
Otaki genkimura	2011	Oct.	22		Db. F.	910	35.57	138.54	[54]
Chichibu-gun									
Mt. Maruyama, Yokoze T.	1995	Oct.	4	7	Db. F.	960	35.59	139.10	[49]
Hiki-gun									
Mt. Kasayama, Ogawa T.	1995	Oct.	14	64	Db. F.	600-820	36.01	139.11	[55]
	2011	Oct.	29						[54]
Kabasaka Pass, Tokigawa T.	1995	Oct.	4	15	Db. F.	820	35.58	139.11	[49]
	2011	Oct.	29	20					[54]
Iimori Pass, Tokigawa T.	1995	Oct.	4	20	Db. F.	780	35.57	139.13	[49]
Hanno City									
Shomaru Pass	1995	Oct.	4	8	Db. F.	680	35.56	139.10	[49]
Koburi Pass	1995	Oct.	15			510	35.56	139.15	[54]
Amamezasu Pass	1995	Nov.	25	2		500	35.55	139.11	Y. K.
Tokyo Metropolis (Okutama Town)									
Mt. Bonomine	1979	Sep.	24	5	*Cr. j* . P	700	35.52	139.09	[39]
	2011	Nov.	3	20	Db. F.	800-900			[54]
Mt. Kuroyama	1979	Oct.	1	2	*Cr. j.* P.	700-900	35.51	139.10	[39]
	2011	Nov.	3			800-900			[54]
Minedani	1980	May	2	96	Db. F.	1,000-1,130	35.50	138.59	[39]

S8.5.2. Brood III (1973±8n Generation: 1973, 1981, 1989, 1997, 2005)

Prefecture / County / Village or Town	Year	Month	Day	Collected individual	Vegetation*	Altitude (m)	Latitude	Longitude	Literatures or collectors*
Nagano Pref.									
Kamiina-gun									
Kayano Hights, Minowa T.	2005	Oct.	2	346	Db. F.	1,000-1,200	35.54	138.02	T.Y.,T.F.,K.N.
Ina City									
Terasawa Experiment Forest,	1973				*L. k* . P.	900-1,000	35.53	138.03	[15]
Shinshu University	1981	Fall							[15]
	1989								[23]
	1997								[23]
	2005	Oct.	1	10					T.Y.,T.F.,K.N.
	2013	Fall							T.Y.,T.F.

S8.5.3. Brood IV (1974±8n Generation: 1982, 1990)

Prefecture / County / Village or Town	Year	Month	Day	Collected individual	Vegetation*	Altitude (m)	Latitude	Longitude	Literatures or collectors*
Shizuoka Pref.									
Shizuoka City									
Mt. Hatanagi	1982	Oct.	11	6		1,100-1,200	35.21	138.10	[9]
Ikawa Experiment Station of Tsukuba University	1990	Oct.		56		1,300	35.21	138.14	M. T., A. T.

*Abbreviations are listed in the last table.

S8.6. Abbreviations of plant and collector.

Vegetation

Conif.	Coniferous
Db.	Deciduous broad-leaved
F.	Forest
Grass	Grassland
P.	Plantation

Plant name

	English	Scientific name
B. spp.	Birch	*Betula* spp.
Cr. j.	Cedar	*Cryptomeria japonica* (L. F.)
		D. Don var. *japonica*
L. k.	Larch	*Larix kaempferi* (Lamb.) Carrire
Q. c.	Oak	*Quercus crispula* Blume

Collector

Initial	Full name
A. T.	Akira Takinami
H. M.	Hiroshi Makihara
H. T.	Hiroshi Tanemura
J. Y.	Jin Yoshimura
K. I.	Kiyoshi Ishii
K. N.	Keiko Niijima
K. Y.	Koji Yamamoto
M. H.	Motohiro Hasegawa
M. K.	Masayuki katakura
M. O.	Mitsuhiro Okada
M. T.	Motoyuki Tsunasaka
N. M.	Nobuko Morihiro
T. F.	Tadashi Fujikawa
T. Y.	Toshio Yoshida
Y. K.	Yukio Kuwabara
Y. M.	Yoshitaka Matsubara
Y. O.	Yasuo Osumi
Oth.	The others
